# HEAL‐D Online: Exploring the potential for the spread and adoption of a virtual culturally tailored diabetes self‐management programme for adults of African and Caribbean heritage

**DOI:** 10.1111/jhn.13396

**Published:** 2024-11-25

**Authors:** Sophie Lowry, Joseph Low, Louise Goff, Sally Irwin, Nick Sevdalis, Pearl Okeke, Oliver Brady, Natasha Curran, Andrew Walker

**Affiliations:** ^1^ Health Innovation Network South London London UK; ^2^ Implementation and Involvement Team National Institute for Health and Care Research Applied Research Collaboration South London London UK; ^3^ Leicester Diabetes Research Centre University of Leicester Leicester UK; ^4^ Centre for Behavioural and Implementation Science Interventions National University of Singapore Singapore

**Keywords:** diabetes, education, EPIS, implementation science, scale‐up, self‐management

## Abstract

**Background:**

People of African and Caribbean heritage in the UK have a higher prevalence of Type 2 Diabetes (T2D) and poorer health outcomes than white Europeans. Healthy Eating and Active Lifestyles for Diabetes Online (HEAL‐D Online) is a co‐designed, culturally tailored T2D self‐management programme for black African and Caribbean adults, which, due to online delivery, is well positioned for spread. This qualitative evaluation uses the Exploration‐Preparation‐Implementation‐Sustainment (EPIS) framework to explore factors affecting scale‐up from delivery and commissioning perspectives.

**Methods:**

Semi‐structured interviews were conducted with nine commissioners and providers of T2D services from three English areas with varying population characteristics to explore scale‐up. Focus groups were held with 15 people of African and Caribbean heritage with T2D lived experience to explore the impact of a digital model of participation. Data were analysed using thematic analysis, with themes mapped onto the EPIS framework exploration phase constructs to consider the outer and inner contextual factors for planning implementation.

**Results:**

Six EPIS constructs were identified by commissioners and providers as key in scaling HEAL‐D Online. People with T2D lived experience explored the online mode of delivery, using the *patient advocacy* construct as the analytical lens. In delivering an online T2D programme, two themes were identified: (1) aligning course content with people's preferences; (2) practicalities to ensure online delivery was acceptable and accessible to the community.

**Conclusions:**

HEAL‐D Online was acceptable with the potential to help address health inequalities. The EPIS framework provided a structure to understand factors in planning scale‐up for an intervention targeting underserved communities.

## Highlights

- Healthy Eating and Active Lifestyles for Diabetes (HEAL‐D) Online is a virtual type 2 diabetes self‐management programme developed to address health inequalities for people of African and Caribbean heritage.
- Findings identified key considerations for planning the scale‐up of HEAL‐D Online based on interviews and focus groups with people from the target population, commissioners and providers of diabetes services in three areas of England using the Exploration‐Preparation‐Implementation‐Sustainment (EPIS) framework.
- These findings expand the currently limited research focused on the Exploration phase of the (EPIS) framework in scaling an intervention aimed at improving outcomes for underserved communities.

## INTRODUCTION

Type 2 Diabetes (T2D) prevalence is estimated to be three times higher in UK African and Caribbean communities than white Europeans,^1^ with onset 10 years earlier^2^ and evidence of poorer health outcomes.^3^, ^4^, ^5^ Healthy Eating and Active Lifestyles for Diabetes (HEAL‐D)^6^ is a culturally tailored, T2D self‐management programme developed to address T2D outcome inequalities among black African and Caribbean adults.^7^ It was co‐designed by researchers, healthcare professionals (including dietitians with relevant experience) and people of African and Caribbean heritage living with T2D. It aims to empower people to self‐manage, offer culturally sensitive and accessible diet and lifestyle advice, and provide a group learning environment to support change.^8^, ^9^

Originally delivered face‐to‐face, and proven highly acceptable,^10^ during the COVID‐19 pandemic HEAL‐D further developed to online delivery. HEAL‐D Online consists of seven live sessions via video call, bringing people together for interactive diet and lifestyle education, physical activity and cooking workshops, underpinned by a peer support and behaviour change ethos. It highlights the importance of nutrition in T2D management, with each session focusing on a different topic such as ‘Taking control! Carbohydrate management’ and ‘Cook and taste: Healthy cooking practices for diabetes self‐management’. Sessions are delivered by a diabetes specialist registered dietitian and a trained community facilitator alongside physiotherapists delivering group‐based physical activity classes. A more detailed description of each session's learning outcomes, content and materials is provided as supplementary information (Additional File 5).

HEAL‐D Online is currently commissioned in South London, UK and delivered by the Nutrition and Dietetics department at Guy's and St Thomas' NHS Foundation Trust. As a virtual programme, it is well positioned for rapid spread. Consequently, in 2021, it was selected for the NHS Insight Prioritisation Programme, which aimed to accelerate the implementation and evaluation of innovation that supports post‐pandemic ways of working in England.^11^

A separate evaluation has shown the feasibility and acceptability of delivering HEAL‐D Online (under review). As part of the next stage of scaling up HEAL‐D Online beyond South London, this study aimed to assess scalability requirements for HEAL‐D Online to the rest of England by using the Exploration‐Preparation‐Implementation‐Sustainment (EPIS) framework.^12^, ^13^ EPIS is a comprehensive implementation framework, developed for public sector contexts to provide a structure to consider the multiple levels and factors affecting implementation at different phases,^12^ but has not previously been used in the scale‐up of a T2D self‐management programme. This preliminary study aimed to explore the perceptions of healthcare commissioners, providers and people of African and Caribbean heritage with T2D about the feasibility of implementing HEAL‐D Online outside South London using the Exploration phase of EPIS. The selection of the EPIS, over other frameworks, was informed by the literature^14^, ^15^, ^16^ against the study aim. EPIS is well suited to look at implementation through a temporal lens, including the key factors associated with the early exploratory stage,^12^, ^14^ which was the focus of this study.

## METHODS

### Design

Qualitative methods using individual semi‐structured interviews and focus groups. This study design has been previously reported in a published study protocol^17^ and is summarised below. Reflexivity was built into the study as a continuous and collaborative process within data collection and analysis.

### Setting and participants

#### Healthcare commissioners/service providers from English integrated care systems (ICSs)

ICSs in England play an important role in bringing together NHS organisations responsible for planning, funding and providing public health and care services to meet local population needs.^18^ Therefore, sampling and recruiting of healthcare commissioners and service providers was based on ICSs. Census 2011 data for England with the 2020–2021 National Diabetes Audit^19^ were used to identify ICSs that represented a mix of urban and rural settings and with a black African and Caribbean population higher, similar or lower than the UK average. Eleven ICSs were selected from a total of 42 ICSs in England. From these 11 ICSs, 26 people with responsibility for commissioning and providing T2D services were identified and invited by email to interview. Nine individuals from three ICSs agreed to participate, representing perspectives from commissioner and service provider organisations (Table 1).

**Table 1:** Study settings and participants.

ICS area	Sample
A. Predominantly urban area in Northwest England with a black African and Caribbean population size greater than the UK average	Service provider = 2 Participant 1 –Diabetes nurse Participant 2 –Diabetes dietitian Commissioner = 5 Participant 4 –Diabetes Commissioning Manager (ICS wide role) Participant 5 –Diabetes Project Manager (ICS wide role) Participant 6 –Commissioning Support Manager (role in an area within ICS) Participant 7 –Commissioning Manager (role in an area within ICS) Participant 8 –Project Manager (role in an area within ICS) People with T2D = 15 Sex: 14 female, 1 male Cultural heritage: 6 African, 5 Caribbean, 1 British, 1 Other, 2 unknown Median age: 65 years Age range: 50–76 years
B. Largely rural area, with some urban districts in the East Midlands with a black African and Caribbean population size similar to the UK average	Service provider = 1 Participant 3 –Diabetes Education Lead
C. Predominantly rural area in Southwest England with a black African and Caribbean population size lower than the UK average	Commissioner = 1 Participant 9 –Diabetes Programme Manager (ICS wide role)

#### People of African and Caribbean heritage

Participant recruitment was coordinated by an African and Caribbean health‐focused community organisation in Northwest England, which identified and invited a convenience sample of 20 people to a community venue, 15 of whom agreed to participate (Table 1).

### Data collection

Data were collected between December 2022 and February 2023.

#### Commissioners and providers

Semi‐structured interviews were conducted with nine participants by SI using topic guides (Additional Files 2 and 3) to explore their perceptions of factors affecting scale‐up – covering existing T2D and online services, health inequalities and local processes. All interviews were recorded and transcribed using Microsoft Teams. SI and SL met regularly during the data collection period to discuss and reflect on the interviews and emerging findings.

#### People with lived experience of T2D

Two focus groups were conducted by PO and JL with 15 participants using a topic guide to explore the facilitators and barriers in delivering an online T2D self‐management programme. This topic guide was developed in collaboration with a lived experience reference group that advised on language and approach (Additional File 4). Both focus groups were audio‐recorded and transcribed verbatim by JL. PO and JL had a debrief discussion following the focus groups.

### Data analysis

All data were initially analysed using thematic analysis to identify key themes.^20^ SL and JL undertook an analysis of the interview and focus group data, respectively. SL independently coded one focus group transcript. SL, SI and JL met regularly to review and ensure reflexivity within their approach to the analysis, which included discussing any discrepancies and considering any personal biases and assumptions. These themes were then mapped onto the EPIS Exploration phase constructs by SL. Findings were reviewed by the wider multi‐disciplinary study team including the lived experience reference group. A key purpose of the study team and lived experience group discussions was to critique and appraise the findings from different perspectives.

## RESULTS

### Factors affecting scale‐up

Six of the Exploration constructs from EPIS were identified as important considerations for planning the scale‐up of HEAL‐D Online (Table 2).

**Table 2:** EPIS Exploration constructs identified as important for planning scale‐up for HEAL‐D Online.

Context	Variables constructs identified and definition^21^
**Outer context:** factors outside of the organisation	**Service environment:** the sociopolitical and economic context that influences the process of implementation and delivery.
	**Funding:** fiscal support provided by the system in which implementation occurs.
	**Patient characteristics:** demographics and individual characteristics of the target population.
**Inner context:** characteristics within an organisation	**Organisational characteristics:** structures or processes that take place and/or exist in organisations that may influence the process of implementation.
	**Patient advocacy:** support or marketing for system change based on consumer needs, priorities and/or demographics.
	**Individual characteristics:** shared or unique characteristics of individuals (e.g. provider, supervisor, director) that influence the process of implementation.

#### Service environment and funding

Commissioners highlighted a lack of national policies supporting the implementation of interventions addressing health inequalities, signifying a lack of a standardised approach. Only one policy was referenced – the NHS England Core20PLUS5 health inequalities framework.^22^ This policy focuses on five clinical areas and does not include T2D, which creates an environment where programmes like HEAL‐D Online risk being deprioritised.

*Funding* was identified as directly aligned with the *service environment* in determining what financial resources were available to allocate to specific areas of need. The ongoing change to commissioning structures in England (i.e., ICSs only being legally established in July 2022) was also highlighted as impacting the availability and allocation of funding for T2D services redesign:

“*A short‐term barrier is the move into the [ICS] because it's complex financially… getting access to [funding] and getting it where I want is a bit tricky*.” – Participant 4, Commissioner

#### Patient characteristics

Participants from commissioner and provider organisations described the importance of understanding the target population to estimate demand and identify inequalities in service access. Some providers suggested that users of existing T2D services do not reflect the wider population in need and that new services should evidence how they will reduce inequality:

“*We're so under‐represented at the groups in terms of ethnic diversity… We do get some South Asian, Middle Eastern and a few African patients, but nothing like the level of in the population with diabetes–it's not representative*.” – Participant 2, Provider

#### Organisational characteristics

Organisational structures and approaches varied across ICSs, with T2D commissioning managed at different levels and by different types of providers. The variability highlighted that mapping local structures is key to developing tailored approaches to implementation.

Commissioners reported that they did not have a specific commissioning framework for T2D structured education. Instead, they used information from professional networks e.g. the national commissioner (i.e. NHS England) clinician networks and reviewed approaches in other areas with a similar population and level of need. This highlighted the importance of peer‐to‐peer connections and the source of the innovation being key factors in T2D commissioning.

ICSs took a data‐driven approach to understand demand and capacity for their local population (e.g. reviewing waiting lists, referral pathways and service gaps). Therefore, new services that address the widest gaps and greatest need are more likely to be identified for implementation:

*“We look at what we are currently commissioning, do waiting list validation, find out where referrals are coming from… We need to understand demand, demographics, preferences of those patients and what will work.”* – Participant 7, Commissioner

Participants in ICS C raised the risk that introducing a new external service (i.e. an online programme delivered by an out‐of‐area provider) could negatively impact existing commissioner‐provider relationships and would require careful consideration of potential costs (such as damaged local relationships) and benefits:

*“If our community services were no longer providing [T2D education] and I went and outsourced that to somebody else, there would be some real relationships to manage because they've been doing this for us for a considerable amount of time.”* – Participant 9, Commissioner

#### Patient advocacy

ICSs reported providing a variety of T2D education services, from nationally accredited courses to locally designed programmes. ICS A offered a programme in 22 languages and ICS B offered a South Asian culturally tailored programme. Despite acknowledging that there was evidenced local demand, none of the ICSs offered or had access to culturally tailored programmes for African and Caribbean communities.

*“We don't have anything specifically for diabetes… We do have a project looking at African and Caribbean communities, but for long term conditions wider.”* – Participant 8, Commissioner

#### Individual characteristics

Some participants raised the potential risk of digital exclusion with online services, although noted this has decreased following the COVID‐19 pandemic. However, participants also identified potential benefits of online delivery, such as increased flexibility and convenience for service providers and service users, financial savings and greater reach and access:

*“There's so many benefits. It improves accessibility. Because we run the courses during the day and evening it reaches a bigger audience including younger people which we were never able to do before. There's less travel for staff and patients. If you've got to travel to a venue, whether it's by car or by bus, and it's six weeks, that's a lot of time and potentially money to some people… And there's a big saving for us on venues.”* – Participant 3, Provider

Providers raised potential challenges around effectively engaging people online compared to face‐to‐face. They also noted that staff might require additional training and support to make online delivery effective. ICS A described undertaking follow‐up calls with patients post in‐person and online delivery of a standard T2D education programme (not targeted at specific communities). They reported that people who attended the online programme appeared to retain fewer key messages:

*“There was no comparison between the knowledge of those who attended face to face and those who attended remotely. It was almost like the remote stuff just went in one ear out the other. So either they weren't listening or they didn't take it in because it wasn't as sensory…”* – Participant 1, Provider

### African and Caribbean people's perspectives on online delivery

*Patient advocacy* was used as the analytical lens, which allowed support for system change to be explored based on consumer needs, priorities and/or demographics. Two key themes were considered a priority by participants when delivering an online T2D programme: (1) course content and (2) practicalities of online delivery.

#### Course content

Participants discussed the topics they wanted to be covered in an online T2D programme, including culturally sensitive dietary advice, exercise options and motivational techniques like goal setting. This aligns with the results from previous co‐design work^7^, ^8^ and indicates content transferability to communities outside of South London.

#### Practicalities of online delivery

There was a general acceptance amongst participants to online delivery, and specifically for a T2D programme such as HEAL‐D Online. Participants felt this acceptance was partly due to an increased use of digital technology following the COVID‐19 pandemic:

*“I already do online programmes. For many people, that's what covid has pushed us into.” –* FG2, Person 2

Several participants highlighted the benefits of online delivery, which echo those raised by providers, such as convenience and greater accessibility. They also acknowledged the risk of digital exclusion and suggested using community settings (e.g., church, cultural or communities groups) where free/subsidised access to technology and support could be provided to mitigate digital exclusion:

“*The problem there is that not everyone can afford to stay online for thirty minutes or one hour, some people use pay as you go, so some people would be excluded*.” – FG2, Person 3

Several participants felt it important to have in‐person contact with other attendees during sessions and contact outside the sessions to provide peer support:

*“If there was a WhatsApp group, people could share things that they have tried with each other.”* – FG1, Person 4

There was also a suggestion of a dedicated ‘support person’ to ensure that people could access the group. This person could identify and resolve technical issues preventing people from attending, and explore wider barriers for not attending and strategies to overcome these:

“*There might be a reason why they couldn't attend. Could be computer problems*.” – FG1, Person 5

## DISCUSSION

Diabetes self‐management, education and support (DSMES) programme are effective – providing improved HbA1c and cardiovascular outcomes and a range of psychological and behavioural benefits.^23^, ^24^, ^25^, ^26^ However, DSMES programmes have lower participation and poorer outcomes for minority ethnicities.^27^, ^28^ DSMES programmes that are culturally tailored to the needs of diverse patients are shown to result in greater improvements in HbA1c, knowledge and quality of life compared to standard training.^29^, ^30^, ^31^ To date, culturally tailored programmes for communities of African and Caribbean heritage have largely been USA‐based, with no such DSMES programmes evaluated in the UK.^29^, ^32^ From the perspective of understanding the potential for scale‐up, this makes drawing any meaningful comparisons challenging due to the significant differences in the way healthcare is commissioned and delivered in the UK and USA.^33^ This preliminary study explores the perceptions of healthcare commissioners, providers and people of African and Caribbean heritage with T2D about the feasibility of implementing HEAL‐D Online in England using the Exploration phase of EPIS.^12^

Commissioners and service providers from three ICSs outside of South London indicated demand for online culturally tailored T2D education courses and perceived them as having the potential in helping to address health inequalities, drive increased education uptake and access, and provide patient‐centred care (with greater flexibility and convenience) and financial savings.

However, several key factors were identified that need to be considered in planning for the implementation of an online T2D education programme: (1) demonstrate to commissioners alignment with the local service environment and funding priorities to access resources for service redesign, (2) utilise data to evidence local need and inequality to justify investment, (3) map local service landscape to understand variability, identify provision gaps and engage service providers and (4) maximise professional networks to share information about online service models.

Implementation would also need to provide additional support and training for staff on delivering online courses effectively. To obtain the required insights and create a system conducive to embedding a programme such as HEAL‐D Online, it will be vital for commissioners and providers to work collectively. A focus on the service environment and appropriate funding regimes, as identified through EPIS, will be key to implementation and sustainability success.^34^

Our preliminary findings indicate that people with African and Caribbean heritage in England who have T2D were familiar with online delivery and considered it an acceptable model of care for T2D education courses. Their preferences for course content and approach to delivery closely aligns with the current HEAL‐D programme, indicating transferability outside South London. A potential barrier is digital exclusion, although working with community groups could help to mitigate this. Addressing health inequalities is the fundamental focus of HEAL‐D; therefore, further consideration must be given to ensuring an online model would not further widen inequality gaps.

From an implementation theory perspective, these findings reinforce the tenet that evidence of effectiveness alone is not sufficient for successful scaled implementation of a programme outside the areas or institutions they originate from.^35^, ^36^, ^37^ The key themes that emerged from providers and commissioners highlighted the importance of locally articulated need and rationale, as well as implementation support strategies that allow the service to be integrated within the local ecosystem – a ‘bolt‐on’ evidenced intervention coming from ‘the evidence’ will simply not work in the studied settings. This supports the idea that the context of implementation for HEAL‐D (i.e., the local circumstances surrounding any implementation effort in the areas we studied, or any area where it might be considered for introduction^38^) will be a major determinant for successful implementation. This explains why several attempts to theorise implementation and scale‐up processes to date, including EPIS, make explicit reference to the context of an implementation effort.^39^ The application of EPIS for this purpose offered a useful and heuristic approach to identifying important contextual elements; these will need to be followed by a mapping to implementation support activities to address the contextual factors identified.^40^

This approach, the study of the implementation context and the subsequent mapping of the identified contextual factors to specific implementation strategies hypothesised to address them are key aspects of a theory‐supported implementation optimisation effort. In addition, the latter phases of EPIS (preparation, implementation and sustainment) could be applied to future research to ensure scale‐up approaches continue to be informed by theory.

### Limitations

The reporting of this study has followed Standards for Reporting Qualitative Research to identify potential limitations and strengths (Additional File 1).

Only two focus groups were conducted, both in ICS A, and most commissioner and service provider interviews were conducted in the same area, limiting the transferability of the findings although it is noted that different areas are likely to face different barriers and facilitators to implementation. In addition, the participating ICSs were self‐selecting, which introduces potential bias in the findings. We were not able to determine the reasons ICSs did not participate, but recognise the pressures health systems are experiencing and the impact this has on people's ability to engage in research activities. Further research is therefore required to explore perceptions of individuals from other areas of England, in order to fully understand our findings' generalisability, and any findings may need to be further explored within local contexts. A recently funded multicentre clinical trial for HEAL‐D will use an embedded process evaluation to explore factors affecting the programme's implementation across different areas using a larger sample. This will significantly add to the evidence about the spread and adoption of HEAL‐D. Nevertheless, findings are consistent with previously published studies^8^ and our parallel service evaluation (in preparation for submission). The number of interviews means it is not possible to compare ICSs to understand contextual differences. A limitation of using EPIS is that there is only one explicit patient perspectives focused construct, and therefore for the focus groups, it was only possible to map to *patient advocacy*.

## CONCLUSIONS

Our study shows that commissioners and providers of T2D services and people with lived experience of T2D consider an online culturally tailored programme for T2D (HEAL‐D Online) as acceptable and has the potential to address health inequalities among people with black African and Caribbean heritage who typically experience poorer health outcomes.

The EPIS framework provided a helpful structure to understand the key factors important for the early phases of planning scale‐up for an intervention aimed at improving outcomes for underserved communities in England.

Through our study, it was noted that further evidence to commissioners and service providers is required for commissioning decisions and implementation. Addressing this, current plans to develop HEAL‐D Online further include a multicentre clinical and cost‐effectiveness trial evaluation^41^ with a continued focus on scale‐up.

## AUTHOR CONTRIBUTIONS

Sophie Lowry conceived and designed the project; designed data collection tools and analysed the data for both qualitative arms; drafted the initial manuscript and gave the final approval of the version to be published. Joseph Low designed the methodology and data collection tools, acquired and analysed the data for the focus groups; reviewed the manuscript critically for important intellectual content and gave the final approval of the version to be published. Louise Goff conceived and designed the project; reviewed the manuscript critically for important intellectual content and gave the final approval of the version to be published. Sally Irwin conceived and designed the project; acquired and analysed the data for the qualitative interviews; reviewed the manuscript critically for important intellectual content and gave the final approval of the version to be published. Nick Sevdalis conceived and designed the project; reviewed the manuscript critically for important intellectual content and gave the final approval of the version to be published. Pearl Okeke designed data collection tools, and acquired and analysed the data for the focus groups; reviewed the manuscript critically for important intellectual content and gave the final approval of the version to be published. Oliver Brady conceived and designed the project; reviewed the manuscript critically for important intellectual content and gave the final approval of the version to be published. Natasha Curran conceived and designed the project; reviewed the manuscript critically for important intellectual content and gave the final approval of the version to be published. Andrew Walker conceived and designed the project; reviewed the manuscript critically for important intellectual content and gave the final approval of the version to be published.Louise Goff, Andrew Walker, Natasha Curran, Oliver Brady and Nick Sevdalis conceived of and proposed the study. Sophie Lowry, Sally Irwin, Joseph Low, and Pearl Okeke conducted data collection and analysis. Sophie Lowry wrote the first draft of the manuscript. All authors contributed edits to the manuscript.The corresponding author attests that all listed authors meet authorship criteria and that no others meeting the criteria have been omitted. The authors read and approved the final manuscript.

## CONFLICTS OF INTEREST STATEMENT

Nick Sevdalis is the director of London Safety and Training Solutions Ltd., which offers training in patient safety, implementation solutions and human factors to healthcare organisations. Louise Goff is involved in the delivery of the HEAL‐D programme that is being evaluated in this research. The other authors have no conflicts of interest to declare.

### PEER REVIEW

The peer review history for this article is available at https://www.webofscience.com/api/gateway/wos/peer-review/10.1111/jhn.13396.

## ETHICS STATEMENT

Ethical clearance for this study was granted by King's College London's Research Ethics Office under the ‘Minimal Risk Registration’ procedure (registration confirmation reference number MRA‐21/22‐28498). All participants provided informed consent to participate.

## TRANSPARENCY DECLARATION

The lead author affirms that this manuscript is an honest, accurate and transparent account of the study being reported. The reporting of this work is compliant with Standards for Reporting Qualitative Research (Additional File 1). The lead author affirms that no important aspects of the study have been omitted and that any discrepancies from the study as planned have been explained (note: the protocol is published and available: https://bmjopen.bmj.com/content/12/11/e067161).

## Supporting information

Supporting information.

Supporting information.

Supporting information.

Supporting information.

Supporting information.

## Data Availability

The datasets used and/or analysed during the current study are available from the corresponding author upon reasonable request.
