# Supplementary material for: HEAL‐D Online: Exploring the potential for the spread and adoption of a virtual culturally tailored diabetes self‐management programme for adults of African and Caribbean heritage
Source: J Hum Nutr Diet. 2024 Nov 25;38(1):e13396. doi: 10.1111/jhn.13396 (PMC11589399; doi:10.1111/jhn.13396)
Supplement: Supplementary file 1 — Supporting information. [file JHN-38-0-s001.pdf]

Additional File 1: Standards for Reporting Qualitative Research (SRQR)

http://www.equator-network.org/reporting-guidelines/srqr/

Title and abstract

	**Page/line no(s). (related to non-anonymised manuscript)**
**Title** - Concise description of the nature and topic of the study Identifying the study as qualitative or indicating the approach (e.g., ethnography, grounded theory) or data collection methods (e.g., interview, focus group) is recommended	Page 1<br>Lines 1-2
**Abstract** - Summary of key elements of the study using the abstract format of the intended publication; typically includes background, purpose, methods, results, and conclusions	Page 2-3<br>Lines 19-45

Introduction

**Problem formulation** - Description and significance of the problem/phenomenon studied; review of relevant theory and empirical work; problem statement	Page 4-5<br>Lines 65-97
**Purpose or research questio**n - Purpose of the study and specific objectives or questions	Page 4-5<br>Lines 89-97

Methods

**Qualitative approach and research paradigm** - Qualitative approach (e.g., ethnography, grounded theory, case study, phenomenology, narrative research) and guiding theory if appropriate; identifying the research paradigm (e.g., postpositivist, constructivist/ interpretivist) is also recommended; rationale	Page 5<br>Line 101
**Researcher characteristics and reflexivity** - Researchers' characteristics that may influence the research, including personal attributes, qualifications/experience, relationship with participants, assumptions, and/or presuppositions; potential or actual interaction between researchers' characteristics and the research questions, approach, methods, results, and/or transferability	Not reported
**Context** - Setting/site and salient contextual factors; rationale	Page 5<br>Lines 103-118
**Sampling strategy** - How and why research participants, documents, or events were selected; criteria for deciding when no further sampling was necessary (e.g., sampling saturation); rationale	Page 5<br>Lines 104-118
**Ethical issues pertaining to human subjects** - Documentation of approval by an appropriate ethics review board and participant consent, or explanation for lack thereof; other confidentiality and data security issues	Page 13<br>Lines 337-340
**Data collection methods** - Types of data collected; details of data collection procedures including (as appropriate) start and stop dates of data collection and analysis, iterative process, triangulation of sources/methods, and modification of procedures in response to evolving study findings; rationale	Page 6<br>Lines 120-132

**Data collection instruments and technologies** - Description of instruments (e.g., interview guides, questionnaires) and devices (e.g., audio recorders) used for data collection; if/how the instrument(s) changed over the course of the study	Page 6<br>Lines 120-132<br>Additional files 2,3,4
**Units of study** - Number and relevant characteristics of participants, documents, or events included in the study; level of participation (could be reported in results)	Page 5<br>Lines 103-118<br>Table 1
**Data processing** - Methods for processing data prior to and during analysis, including transcription, data entry, data management and security, verification of data integrity, data coding, and anonymization/de-identification of excerpts	Page 6<br>Lines 121-132
**Data analysis** - Process by which inferences, themes, etc., were identified and developed, including the researchers involved in data analysis; usually references a specific paradigm or approach; rationale	Page 6<br>Lines 133-139
**Techniques to enhance trustworthiness** - Techniques to enhance trustworthiness and credibility of data analysis (e.g., member checking, audit trail, triangulation); rationale	Page 6<br>Lines 135-139

Results/findings

**Synthesis and interpretation** - Main findings (e.g., interpretations, inferences, and themes); might include development of a theory or model, or integration with prior research or theory	Pages 6-10<br>Lines 140-251
**Links to empirical data** - Evidence (e.g., quotes, field notes, text excerpts, photographs) to substantiate analytic findings	Pages 6-10<br>Lines 140-251<br>Quotes throughout

Discussion

**Integration with prior work, implications, transferability, and contribution(s) to the field -** Short summary of main findings; explanation of how findings and conclusions connect to, support, elaborate on, or challenge conclusions of earlier scholarship; discussion of scope of application/generalizability; identification of unique contribution(s) to scholarship in a discipline or field	Pages 10-11<br>Lines 253-294
**Limitations** - Trustworthiness and limitations of findings	Page 11-12<br>Lines 295-304

Other

**Conflicts of interest** - Potential sources of influence or perceived influence on study conduct and conclusions; how these were managed	Page 13<br>Lines 346-350
**Funding** - Sources of funding and other support; role of funders in data collection, interpretation, and reporting	Page 13<br>Lines 351-355

Reference:

O'Brien BC, Harris IB, Beckman TJ, Reed DA, Cook DA. **Standards for reporting qualitative research: a synthesis of recommendations.** *Academic Medicine*, Vol. 89, No. 9 / Sept 2014 DOI: 10.1097/ACM.0000000000000388
