# Supplementary material for: HEAL‐D Online: Exploring the potential for the spread and adoption of a virtual culturally tailored diabetes self‐management programme for adults of African and Caribbean heritage
Source: J Hum Nutr Diet. 2024 Nov 25;38(1):e13396. doi: 10.1111/jhn.13396 (PMC11589399; doi:10.1111/jhn.13396)
Supplement: Supplementary file 2 — Supporting information. [file JHN-38-0-s005.pdf]

Additional File 2: Service Provider Topic Guide

Pilot Site commissioner and service provider Interviews

Interview Topic Guide – Service providers/professionals

Introduction

Hi, I am [INTRODUCE SELF]

Before we start can I please confirm your name?

Purpose of the discussion:

The Health Innovation Network, south London Applied Research Collaborative and Kings College London are collaborating on a project to explore the scale up potential of HEAL-D which is an online, culturally tailored, self-management diabetes education programme. The programme is for adults with type 2 diabetes of African and Caribbean heritage.

The purpose of this conversation is to help us to gain insight into your local commissioning and operational structures and to understand different factors and challenges that may affect the scale-up of programmes such as HEAL-D online.

We will ask you some questions about your local commissioning and provider pathways. Where possible, please focus on the commissioning and delivery of type 2 diabetes structured education courses.

Consent:

We provided you with a document giving background information about the HEAL-D programme, the NIPP project, the purpose of this discussion and how we will use the information you provide. Do you have any questions about this?

Please confirm that you agree with the following statements

- I have read and understood the information and required consent as set out in this information sheet.
- I consent to using Microsoft Teams to complete the interview (optional – recording / transcription are not compulsory)
- I consent to the use of anonymised information provided for the purposes outlined above
- I understand that I can withdraw consent at any time.

Check whether participant has any questions and is happy to begin the interview.

<table>
<tr><td colspan="2">START RECORDING AND TRANSCRIPTION (IF APPLICABLE)

I am going to take some notes throughout our conversation, so you may hear some typing.

I also may need to ask you to pause briefly whilst I write up any key points.</td></tr>
<tr><td>Section 1 – Provision of diabetes services</td><td></td></tr>
<tr><td>What type 2 diabetes structured education courses do you currently deliver?
Are these online / in person?
If online, are these remote delivery (I.e. trainers delivering a course) or a digital access course?
Do you offer any culturally tailored courses?
[IF <u>HAVE</u> DELIVERED ONLINE COURSES]

Thinking about online courses specifically, what are the key considerations to make it successful?
What challenges have you come across when delivering virtual courses?
From your perspective, how did you overcome these challenges?
What benefits does offering virtual courses provide to your service delivery model and to service users?
[IF <u>HAVEN’T</u> DELIVERED ONLINE COURSES]

Thinking about online courses specifically, what are the key considerations to make it successful?
What challenge do you think there may be in delivering virtual courses?
From your perspective, how would you overcome these challenges?
What benefits do you believe offering virtual courses would provide to your service delivery model and to service users?</td><td></td></tr>
<tr><td>Section 2 – Health Inequalities</td><td></td></tr>
<tr><td>Are you aware of your local population and local need in relation to type 2</td><td></td></tr>
</table>

diabetes education?<br><br>• Do you feel you are meeting this need? Why / how?<br><br>Do you experience any common themes in non-attendance and attrition/drop out to type 2 diabetes structured education?<br><br>• What themes?	
**Section 3 – Learning**	
What key things about a programme gain buy in from providers to engage with training and delivery?<br><br>• Explore: accreditation, CPD, support, what do they want to achieve?<br><br>From your experience, what would facilitate improved commissioning and provision of type 2 diabetes structured education?<br><br>• Are there any lessons we could learn from other service area commissioning?	
**Closing remarks**	
Is there anything else that you believe would be important to consider / helpful for our work?	
**Thank you and close**	
That is the end of our discussion. Thank you for your participation.<br><br>Do you have any questions?<br><br>I will now stop the recording (if applicable)<br><br>**STOP RECORDING AND TRANSCRIPTION (IF APPLICABLE)**	
