# Supplementary material for: HEAL‐D Online: Exploring the potential for the spread and adoption of a virtual culturally tailored diabetes self‐management programme for adults of African and Caribbean heritage
Source: J Hum Nutr Diet. 2024 Nov 25;38(1):e13396. doi: 10.1111/jhn.13396 (PMC11589399; doi:10.1111/jhn.13396)
Supplement: Supplementary file 3 — Supporting information. [file JHN-38-0-s002.pdf]

Additional File 3: Commissioner Topic Guide

Pilot Site Stakeholder Interviews

Interview Topic Guide - Commissioners

Introduction

Hi, I am [INTRODUCE SELF]

Before we start can I please confirm your name?

Purpose of the discussion:

The Health Innovation Network, south London Applied Research Collaborative and Kings College London are collaborating on a project to explore the scale up potential of HEAL-D which is an online, culturally tailored, self-management diabetes education programme. The programme is for adults with type 2 diabetes of African and Caribbean heritage.

The purpose of this conversation is to help us to gain insight into your local commissioning and operational structures and to understand different factors and challenges that may affect the scale-up of programmes such as HEAL-D online.

We will ask you some questions about your local commissioning and provider pathways. Where possible, please focus on the commissioning and delivery of type 2 diabetes structured education courses.

Consent:

We provided you with a document giving background information about the HEAL-D programme, the NIPP project, the purpose of this discussion and how we will use the information you provide. Do you have any questions about this?

Please confirm that you agree with the following statements

- I have read and understood the information and required consent as set out in this information sheet.
- I consent to using Microsoft Teams to complete the interview (optional – recording / transcription are not compulsory)
- I consent to the use of anonymised information provided for the purposes outlined above
- I understand that I can withdraw consent at any time.

Check whether participant has any questions and is happy to begin the interview.

<table>
<tr><td colspan="2">

START RECORDING AND TRANSCRIPTION (IF APPLICABLE)

I am going to take some notes throughout our conversation, so you may hear some typing.

I also may need to ask you to pause briefly whilst I write up any key points.

</td></tr>
<tr><td>

Section 1 – Commissioning of diabetes services

</td><td></td></tr>
<tr><td>

How are Type 2 diabetes structured education services currently commissioned in your area?

- Are services commissioned locally / place based / by Local Care Partnerships? Or ICB wide?
- Do you commission places or courses?
- Are your existing services online or in person?
- What is the referral pathway for patients to be referred to a type 2 diabetes structured education programme?

What do you look for when commissioning/recommissioning type 2 diabetes structured education?

- Do you follow a diabetes commissioning framework? If yes, what are the key requirements?
- (explore what is decision making based on – framework, cost, evidence of clinical effectiveness, feasibility, service user feedback, local population health data, health inequalities)?
- How do you find out about new ideas and innovative services?
- What are the barriers to introducing a new service?

</td><td></td></tr>
<tr><td>

Section 2 – Health Inequalities

</td><td></td></tr>
<tr><td>

How do you account for health inequalities in diabetes commissioning?

- Do you follow any health inequalities frameworks or guidance?
- Do you know who the groups are with unequal access or unequal health outcomes, in relation to type 2 diabetes, in your population?
- Are you aware of any targeted interventions/anything specific for people of African/Caribbean heritage or other cultural needs?

</td><td></td></tr>
</table>

**Section 3 – Online services**	
Do you currently offer or are you considering any online or virtual structured education type 2 diabetes services?<br><br>• If yes:<br>○ Can you explain how this was commissioned?<br>○ What worked well about this process?<br>○ What challenges did you encounter? And from a commissioning perspective, how have you overcome these challenges?<br>○ From your experience, how would you approach commissioning similar courses in the future?<br>○ What benefits do you believe virtual courses provide to commissioners?<br>• If no:<br>○ Why not? (reasons for not commissioning/challenges)<br>○ What do you believe are some of the opportunities online services offer? (explore how remote HEAL-D could support areas of low population density)<br>○ What would facilitate you to commission online structured education?	
**Section 4 – Learning**	
From your experience, what would facilitate improved commissioning and provision of type 2 diabetes structured education?<br><br>• Are there any lessons we could learn from other service area commissioning?	
**Closing remarks**	
Is there anything else that you believe would be important to consider / helpful for our work?	
**Thank you and close**	
That is the end of our discussion. Thank you for your participation.<br><br>Do you have any questions?<br><br>I will now stop the recording (if applicable)	

**STOP RECORDING AND TRANSCRIPTION (IF APPLICABLE)**	
