# Supplementary material for: HEAL‐D Online: Exploring the potential for the spread and adoption of a virtual culturally tailored diabetes self‐management programme for adults of African and Caribbean heritage
Source: J Hum Nutr Diet. 2024 Nov 25;38(1):e13396. doi: 10.1111/jhn.13396 (PMC11589399; doi:10.1111/jhn.13396)
Supplement: Supplementary file 4 — Supporting information. [file JHN-38-0-s003.pdf]

Additional File 4: Focus Group Session Plan and Topic Guide

Registration & refreshments

Aim: make people feel welcome and comfortable

Welcome & housekeeping (5 minutes)

Aim: cover logistics and set ground rules for the day

Introduction (5 minutes)

Aim: ensure everyone understands the purpose of the session

Ice breaker (5 minutes)

Aim: make people feel comfortable and learn a little bit about why everyone decided to join the focus group

In pairs discuss the following questions, then share your partners answers to the group

- What’s your name?
- How long have you / your relative been living with type 2 diabetes?
- Have you got any experience of attending a diabetes self-management course?

Go round group and ask everyone to share their name.

Main activity (1hr - 1hr15m)

Aim: discuss perspectives about the provision of online learning in order to understand perceptions of the potential acceptability and implications of a digital model of participation.

1. **How would you feel if you were asked to attend a series of online sessions specifically designed to help manage your diabetes?**

Prompts (issues to cover if not mentioned):

- What are your preferences around accessing a self-management course online versus face to face, and why?
- What are the *benefits of attending an online course*?
- What are the *challenges of attending an online course?*
- *What would encourage you to attend*?
- Who would you trust to inform you about the course?
- What device(s) / technology could you use to attend the session? Can you see any challenges with this?

2. **Thinking about HEAL-D specifically, with HEAL-D being delivered over 7**

**weeks, at regular one-week time slots, such as Wednesday at 5pm**<br>• How would the HEAL-D schedule work for you?<br>• Would the fact it is online make it more/less easy for you to attend? Why?<br>• A key element of HEAL-D is that it is live and interactive. *How do you think being online might affect the way you interact with peers / facilitators?*<br>• If we were planning on rolling out HEAL-D Online outside south London, what other issues would we need to consider?<br><br>**3. Finally, is there anything else we have not discussed today that you would like to talk about / mention?**
**Closing remarks (5 minutes)**<br><br>Aim: make people feel listened to and valued. Provide key reflections and let them know what will happen next.
**Close**

2
