# Supplementary material for: HEAL‐D Online: Exploring the potential for the spread and adoption of a virtual culturally tailored diabetes self‐management programme for adults of African and Caribbean heritage
Source: J Hum Nutr Diet. 2024 Nov 25;38(1):e13396. doi: 10.1111/jhn.13396 (PMC11589399; doi:10.1111/jhn.13396)
Supplement: Supplementary file 5 — Supporting information. [file JHN-38-0-s004.pdf]

Additional File 5: The HEAL-D Online programme - session learning outcomes, content and materials

Learning objectives	Content and materials
**1. Diabetes – It’s in your hands! An introduction to T2D self-management**	
Understand what T2D is and how it develops.<br>Understand the role of insulin, glucose and blood glucose in health and T2D.<br>Be introduced to key ways in which diet and lifestyle influence T2D.<br>Understand the principles of a healthy, balanced diet.<br>Understand how to set personal goals.	*Diabetes – It’s in your hands* slide set<br>*Getting Started* information booklet<br>*Diabetes Explained* information booklet<br>*Balanced Diet* information booklet<br>*Diabetes Explained* animation film<br>*Diabetes True/False* interactive group activity<br>*Our Objectives* interactive group activity<br>*Tree of Life* interactive group activity<br>*Measuring Your Progress* form
**2. Get Moving!**	
Understand the importance of physical activity in T2D management.<br>Know the physical activity recommendations for T2D management.<br>Understand the difference between strength training and aerobic exercise and why they are important in T2D.<br>Review HEAL-D activity targets and set personal physical activity goals.<br>Explore personal barriers to engagement in physical activity.<br>Know what physical activity provision is available locally.<br>Learn about the HEAL-D physical activity resources.<br>Explore problem-solving and action planning techniques.<br>Participate in a group-based physical activity session.	*Get Moving!* slide set<br>*Get Moving!* information booklet<br>*Overcoming Barriers* interactive group discussion<br>*Get Moving!* patient stories film<br>*Step It Out* homework card<br>Pedometer<br>HEAL-D home exercise videos & exercise band<br>Virtual group exercise session – Resistance bands
**3. Taking Control!**	
Know different types of carbohydrate and food sources of these.<br>Understand how carbohydrate foods affect blood glucose.<br>Know carbohydrate content of commonly consumed foods.<br>Know appropriate portion sizes of carbohydrate foods.<br>Review the role of insulin in blood glucose control.<br>Participate in a group-based physical activity session.	*Step It Out* homework feedback<br>*Taking Control!* slide set<br>*Taking Control!* information booklet<br>*Identifying Carbohydrates* group discussion<br>*Taking Control!* patient stories film<br>*Sugar Cube Game* interactive group activity<br>*Overcoming Barriers* interactive group discussion<br>Carbohydrate portion guide handout<br>*Take Control* homework card<br>Virtual group exercise session – dance aerobics

**4. Shape Up!**	
Understand how weight and shape influence T2D.<br>Understand that reducing waist circumference is beneficial for T2D management.<br>Learn about and experience mindful eating.<br>Participate in a group-based physical activity session.	*Take Control* homework feedback<br>*Shape Up!* slide set<br>*Shape Up!* information booklet<br>*Middle Measurements* interactive group activity<br>*Mindful Eating* homework card<br>Tape measures<br>Virtual group exercise session – Circuit training
**5. Drop the Pressure!**	
Understand how salt and saturated fat affect cardiovascular health.<br>Understand what foods monounsaturated fats and n-3 polyunsaturated fats are found in.<br>Understand the importance of vegetables for heart health.<br>Know the salt content of common foods and hidden sources of salt in the diet.<br>Understand the importance of physical activity and weight management for heart health.<br>Participate in a group-based physical activity session.	*Mindful Eating* homework feedback<br>*Drop the Pressure!* slide set<br>*Drop the Pressure!* information booklet<br>*Finding Salt* interactive group activity<br>*Try a New Activity* homework card<br>Virtual group exercise session – flex & stretch
**6. Putting it into Practice: cook & taste**	
Learn techniques for cooking with less salt and oil.<br>Learn techniques for adding extra vegetables into the diet.<br>Learn techniques for reducing saturated fat in the diet.<br>Learn techniques for reducing carbohydrate portion sizes.<br>Take part in a cook and taste session.<br>Taste foods/dishes prepared with reduced salt and fat.<br>Taste foods/dishes prepared with increased vegetables.	*Try a New Activity* homework feedback<br>*Putting it into Practice* information booklet<br>Virtual cooking stations for increasing vegetables, low fat/healthy fat, reducing salt, reducing carbohydrate, increasing fibre.<br>Preparing low salt seasonings station.<br>*Diabetes Friendly Cooking* homework card
**7. Plans for Life**	
Reflect on successes of HEAL-D programme.<br>Identify situations which may cause lapses.<br>Identify tools to manage challenging self-management situations.<br>Participate in a group-based physical activity session.	*Diabetes Friendly Cooking* homework feedback<br>*Plans for Life* slide set<br>*What We've Learnt* interactive group quiz<br>*Managing Lapses* interactive group discussion<br>*Celebrating Successes* interactive group discussion<br>HEAL-D certificates of participation<br>Group exercise session – Zumba™
